# Massive Consumption of Gelatinous Plankton by Mediterranean Apex Predators

**DOI:** 10.1371/journal.pone.0031329

**Published:** 2012-03-21

**Authors:** Luis Cardona, Irene Álvarez de Quevedo, Assumpció Borrell, Alex Aguilar

**Affiliations:** IRBIO and Department of Animal Biology, Faculty of Biology, University of Barcelona, Barcelona, Spain; Institut Pluridisciplinaire Hubert Curien, France

## Abstract

Stable isotopes of carbon and nitrogen were used to test the hypothesis that stomach content analysis has systematically overlooked the consumption of gelatinous zooplankton by pelagic mesopredators and apex predators. The results strongly supported a major role of gelatinous plankton in the diet of bluefin tuna (*Thunnus thynnus*), little tunny (*Euthynnus alletteratus*), spearfish (*Tetrapturus belone*) and swordfish (*Xiphias gladius*). Loggerhead sea turtles (*Caretta caretta*) in the oceanic stage and ocean sunfish (*Mola mola*) also primarily relied on gelatinous zooplankton. In contrast, stable isotope ratios ruled out any relevant consumption of gelatinous plankton by bluefish (*Pomatomus saltatrix*), blue shark (*Prionace glauca*), leerfish (*Lichia amia*), bonito (*Sarda sarda*), striped dolphin (*Stenella caerueloalba*) and loggerhead sea turtles (*Caretta caretta*) in the neritic stage, all of which primarily relied on fish and squid. Fin whales (*Balaenoptera physalus*) were confirmed as crustacean consumers. The ratios of stable isotopes in albacore (*Thunnus alalunga*), amberjack (*Seriola dumerili*), blue butterfish (*Stromaeus fiatola*), bullet tuna (*Auxis rochei*), dolphinfish (*Coryphaena hyppurus*), horse mackerel (*Trachurus trachurus*), mackerel (*Scomber scombrus*) and pompano (*Trachinotus ovatus*) were consistent with mixed diets revealed by stomach content analysis, including nekton and crustaceans, but the consumption of gelatinous plankton could not be ruled out completely. In conclusion, the jellyvorous guild in the Mediterranean integrates two specialists (ocean sunfish and loggerhead sea turtles in the oceanic stage) and several opportunists (bluefin tuna, little tunny, spearfish, swordfish and, perhaps, blue butterfish), most of them with shrinking populations due to overfishing.

## Introduction

An interest in gelatinous plankton has developed over the past decades after a long period of neglect by marine biologists [1]. The driver of this change is the widespread perception that the abundance of medusa and ctenophores is increasing in many oceanic basins [2], [3], [4] and the concern about the potential negative impact of these phenomena on commercially important fisheries [2] and the tourism industry [5].

Avian and Rottini-Sandrini (1988) [6] and Harbison (1993) [7] were the first to propose that a large number of pelagic predators may opportunistically consume gelatinous zooplankton and suggested that overfishing would release salps, ctenophores and medusa from tight predator control. The proliferation of gelatinous plankton in several heavily fished regions might be considered to support such a hypothesis, but available evidence indicates that competitive release, and not the relaxation of top-down control, is the most likely mechanism [8], [9], [10]. As a consequence, overfishing of gelatinous plankton consumers is presented in recent reviews as a plausible hypothesis but with little direct supporting evidence [4], [5].

Central to the top-down relaxation hypothesis is the hypothetical existence of a large community of pelagic predators that may opportunistically consume gelatinous plankton, thereby stabilizing their populations [6], [7]. Although there is increasing evidence that many pelagic fish may occasionally consume gelatinous plankton [11], and some ecosystem models include tuna and billfish as major consumers of gelatinous plankton [12], it is a big leap from an occasional-consumption model to the strong top-down control assumed by the top-down relaxation hypothesis. Furthermore, nothing is known about the actual significance of gelatinous plankton in the diet of most pelagic mesopredators and apex predators, and there is hard evidence for massive consumption of gelatinous plankton only for some fishes [7], [13], [14] and pelagic sea turtles [15].

Massive proliferations of gelatinous plankton in the Mediterranean have raised considerable public interest [6], [16]–[19]) because of their potential impact on the tourism industry. Outbreaks in the region are known to be tightly linked to climatic variability [16], [20], [21], and those of the pink jellyfish (*Pelagia noctiluca*) have been recorded for almost two centuries. Nevertheless, predator release due to overfishing has been repeatedly suggested as a potential factor in the jellyfish proliferations in the region [6], [10], [18], [19], [22].

Stomach content analysis has revealed the consumption of gelatinous plankton by several Mediterranean species of pelagic mesopredators and apex predators [23]–[31], most of them targeted or incidentally bycaught by commercial fisheries [32], [33]. Although the demographic trajectories of most of these populations are unknown, the populations of loggerhead sea turtles migrating into the Mediterranean from Atlantic nesting beaches (*Caretta caretta*) and those of swordfish (*Xiphias gladius*) and bluefin tuna (*Thunnus thynnus*) of the eastern Atlantic stock spawning into the Mediterranean have undergone relevant declines over the past few decades [34]–[36]. This scenario would support the top-down relaxation hypothesis, although gelatinous plankton always occur in very low numbers in the stomach contents of Mediterranean predators. Whether this is because of their fragility and difficulty of identification [11] or whether it reveals that the dietary significance is truly minor remains unknown. This paper aims to answer this question through stable isotope analysis, as the ratios of stable isotopes in gelatinous zooplankton are different from those of other potential prey [37]–[39] and previous studies have demonstrated the utility of this method for assessing the dietary relevance of gelatinous zooplankton in the diet of marine vertebrates [25].

## Materials and Methods

### Ethics

All of the species sampled were caught for purposes other than research, except jellyfishes, salps, hyperiidean amphipods and euphausiids. No specific approval is required in Spain to undertake research on samples supplied by official channels and coming from by-catch of commercial fishing vessels. Loggerhead turtles, fin whales and bottlenose dolphins are protected by Spanish laws and hence samples were collected by the Marine Animals Recovery Center (CRAM), the organism officially designated by the Catalonian regional government to collect stranded marine animals, undertake necropsies and distribute samples among research groups.

### Study site and sample collection

Samples were collected from 2006 to 2007 in the northwestern Mediterranean, between the Iberian Peninsula and the Balearic islands. The area has supported very dense populations of gelatinous plankton since 2003, with pink jellyfish (*Pelagia noctiluca*) being present year round. Pelagic mesopredators (blue butterfish (*Stromateus fiatola*), bullet tuna (*Auxis rochei*), horse mackerel (*Trachurus trachurus*), mackerel (*Scomber scombrus*) and pompano (*Trachinotus ovatus*)) and apex predators (albacore (*Thunnus alalunga*), amberjack (*Seriola dumerili*), bluefin tuna (*Thunnus thynnus*), bluefish (*Pomatomus saltatrix*), blue shark (*Prionace glauca*), bonito (*Sarda sarda*), dolphinfish (*Coryphaena hippurus*), fin whale (*Balaenoptera physalus*), leerfish (*Lichia amia*), little tunny (*Euthynnus alletteratus*), loggerhead sea turtles (*Caretta caretta*), striped dolphin (*Stenella caeruleoalba*), swordfish (*Xiphias gladius*) and spearfish (*Tetrapturus belone*)) were captured by commercial fishing vessels operating in the area, and tissue samples of these species were collected by observers aboard. Fin whales and striped dolphins were the only exception, as dead, stranded individuals were sampled.

Potential prey were also sampled from the catch of commercial vessels operating in the same area (anchovy (*Engraulis encrasicolus*), horse mackerel (*Trachurus trachurus*), lanternfish (*Lampanyctus crocodilus*), longfin squid (*Loligo vulgaris*), mackerel (*Scomber scombrus*), sardine (*Sardina pilchardus*) and shortfin squid (*Todarodes sagittatus*)), whereas gelatinous plankton (fried egg jellyfish (*Cotylorhiza tuberculata*), pink jellyfish (*Pelagia noctiluca*) and salps (*Salpa maxima*)) and hyperiidean amphipods were collected with a dip net during the fishing operations. Euphausiids (*Meganyctiphanes norvegica*) were collected from the stomach contents of bullet tuna, and a plankton-net was used to collect copepods.

White dorsolateral muscle was sampled from all fish, as well as mantle from the cephalopods and carapace scutes from loggerhead sea turtles. Gelatinous plankton and crustaceans were fully homogenized. All of the species had a sample size of 5, except for blue butterfish, and copepod, hyperiidean and krill samples were collective. Samples were stored at −20°C prior to analysis.

### Stable isotope analysis

Once thawed, tissues were dried at 60°C and ground to a fine powder, and their lipids were then extracted with a chloroform/methanol (2∶1) solution. Crustacean samples were split in two subsamples. One of them was treated with O.5 N ClH to remove the inorganic carbonates of the skeleton and avoid any bias in the δ^13^C. However, acidification may modify the relative concentration of N isotopes, so the other subsample was used to determine the δ^15^N value. All of the samples were weighed into tin cups, combusted at 1,000°C, and analyzed in a Flash 1112 IRMS Delta C Series EA Thermo Finnigan continuous flow isotope ratio mass spectrometer. A Carlo Erba Flash 112 elemental analyzer coupled to the isotope ratio mass spectrometer was used to measure the % C and % N of the dry weight. Stable isotope abundances were expressed in δ notation according to the following expression:
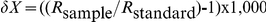
where *X* was ^13^C or ^15^N and *R*_sample_ and *R*_standard_ were the corresponding ratio ^13^C/^12^C or ^15^N/^14^N of the sample and the standard. The standards for ^13^C and ^15^N were Vienna Pee Dee Belemnite (VPDB) and atmospheric nitrogen (air), respectively. International isotope secondary standards for carbon (IAEA CH_6_ (δ^13^C = −10.4‰), USGS 24 (δ^13^C = −16.1‰), IAEA CH_7_ (δ^13^C = −31.8‰)) were used to a precision of 0.2‰, and for nitrogen (IAEA NO_3_ (δ^15^N = +4.7‰), IAEA N_2_ (δ^15^N = +20.3‰), IAEA N_1_ (δ^15^N = +0.4‰)) to a precision of 0.3‰.

### Energy density

The proximate chemical composition of pink jellyfish, salps, mackerels and longfin squids was assessed to determine energy density. Once thawed, samples were weighed and dried at 100°C until a constant weight was reached. The moisture content was calculated by gravimetric difference between wet and dry mass [40]. Dry samples were homogenized and a subsample burnt for six hours in a muffle furnace at 600°C for ash determination [41]. A second subsample was processed to determine its nitrogen content by means of an elemental analyzer, a value that was later multiplied by a conversion factor of 5.8 to obtain the relative abundance of proteins in the dry material [42], [43]. A third subsample was processed to determine its lipid content. Lipids were extracted with a chloroform/methanol (2∶1) solution [44] and their content was determined by the gravimetric difference between fat and non-fat dry mass. Protein and lipid contents were converted to energy density using the mean combustion equivalents reported by [43], i.e., 23.9 kJ g^−1^and 39.5 kJ g^−1^ respectively. Carbohydrate content was not measured, as is low in fishes and jellyfishes and has a practically negligible contribution to their energy density [40]. In the case of salps, tunica is though to have a low digestibility for vertebrates [45].

### Data analysis

ANOVA and a Tukey post-hoc test, conducted with the PASW 17 software package, were used to test differences in the concentrations of stable isotopes of potential prey. As SIAR requires that the variability associated with sources is normally distributed [45], normality was assessed for each group using Lilliefors test.

The Bayesian mixing model SIAR (Stable Isotope Analysis in R) [46] was used to calculate the relative contribution of the potential preys to the diet of each focal species. Bayesian approaches allow for the incorporation of not only isotopic values, elemental concentrations and diet-tissue isotopic discrimination factors within the mixing models but also the uncertainties involved in all these values, and so provide results that are expected to be considerably more robust when it comes to quantifying feeding preferences when compared with those in previous modeling approaches [46]–[48]. Furthermore, as the resulting posterior distributions of the proportions of various sources within the diet of a consumer have associated probabilities, it is possible to use the most likely solution as a single metric for a given dietary component in subsequent analyses [47], [48].

The model parameters were the following: the isotope ratios and the elemental concentrations of the potential food sources, the isotope ratio of tissue and the trophic shift, or isotopic enrichment, for carbon and nitrogen from prey to predator. Prey-to-predator isotopic enrichment for fishes, mammals and loggerhead sea turtles were taken from Reich et al. (2008) [49] and Caut et al. (2009) [50]. Published data on stomach contents were used to identify potential preys other than gelatinous plankton.

Although SIAR incorporates uncertainty about diet-tissue isotopic discrimination factors in the form of standard deviation, we conducted a sensitivity analysis running SIAR for bluefin tuna with diet-tissue isotopic discrimination factors ranging from 1.1 to 2.3‰ for δ^13^C and from 2.2 to 3.4‰ and δ^15^N.

Data are usually shown as mean ± standard deviation (SD), but the feasible contribution of potential prey species to the diet is reported as the mean and 95% credibility interval.

## Results

**Figure 1:**
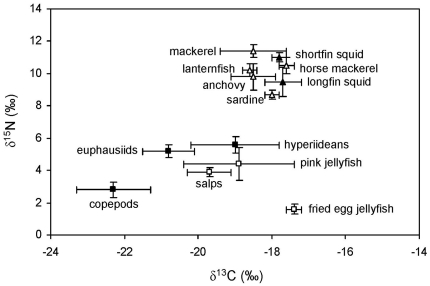
Stable isotope ratios in the potential prey of apex predators from the northwestern Mediterranean. Potential prey considered: pelagic crustaceans (solid squares), gelatinous plankton (empty squares), squid (solid triangles) and small pelagic and mesopelagic fish (empty triangles). Error bars show standard deviation.

Table 1 summarizes the sample size and stable isotope ratios of all the species analyzed. Figure 1 shows the pelagic isoscape of the northwestern Mediterranean. Differences in the δ^13^C and δ^15^N of the potential prey were statistically significant (ANOVA; δ^13^C: F_12,52_ = 26.577, p<0.001; δ^15^N: F_12,52_ = 224.311, p<0.001). Nine groups of potential prey differing in the concentration of at least one stable isotope existed, on the basis of Tukey post-hoc tests: fried egg jellyfish, pink jellyfish, salps, copepods, euphausiids, hyperiideans, sardine, other small pelagic fish and squid (anchovy, horse mackerel, lanternfish and longfin squid) and midsize pelagic fish and squid (mackerel and shortfin squid). Data were normally distributed within all the groups and hence these groups were later used for running SIAR, although the δ^13^C of copepods and fried egg jellyfish were so distinct from those of the focal species (see below) that they were no longer considered as potential prey.

**Table 1:** Sample size and stable isotope ratios of pelagic prey and predators in the western Mediterranean Sea.

Species	Common name	n	δ13 C		δ15 N	
			mean	±SD	mean	± SD
**Prey**						
*Copepoda*	Copepods	A	−22.3	1.0	2.8	0.5
*Cotylorhiza tuberculata*	Fried egg jellyfish	5	−17.4	0.2	1.6	0.3
*Engraulis encrasicolus*	European anchovy	5	−18.5	0.6	9.8	0.8
Hyperiidae	Hyperideans	A	−19.0	1.2	5.6	0.5
*Lampanyctus crocodilus*	Jewel lanternfish	5	−18.6	0.2	10.2	0.4
*Loligo vulgaris*	European common squid	5	−17.7	0.5	9.5	0.9
*Meganyctiphanes*	Krill	A	−20.8	0.7	5.2	0.4
*Pelagia noctiluca*	Pink jellyfish	5	−17.8	0.6	5.6	0.5
*Sardina pilchardus*	European pilchard	5	−18.0	0.2	8.7	0.2
*Salpa maxima*	Salp	5	−19.7	0.6	3.9	0.3
*Todarodes sagittatus*	European flying squid	5	−17.8	0.1	11.0	0.1
**Predators**						
*Auxis rochei*	Bullet tuna	5	−18.1	0.3	9.5	0.5
*Balaenoptera physalus*	Fin whale	5	−18.4	0.1	8.7	0.1
*Caretta caretta* (neritic stage)	Loggerhead sea turtle	5	−16.3	0.4	10.1	1.7
*Caretta caretta* (pelagic stage)	Loggerhead sea turtle	5	−17.6	0.2	6.7	0.4
*Coryphaena hippurus*	Dolphinfish	5	−18.3	0.3	9.8	0.7
*Euthynnus alletteratus*	Little tunny	5	−17.2	0.1	10.4	0.4
*Lichia amia*	Leerfish	5	−17.1	0.3	13.1	1.0
*Mola mola*	Sunfish	5	−17.6	0.5	7.7	0.4
*Pomatomus saltatrix*	Bluefish	5	−16.9	0.3	14.8	0.4
*Prionace glauca*	Blue shark	5	−17.2	0.7	13.3	0.4
*Sarda sarda*	Atlantic bonito	5	−16.8	0.3	12.8	1.2
*Scomber scombrus* *	Mackerel	5	−18.5	0.9	11.4	0.4
*Seriola dumerili*	Amberjack	5	−17.7	0.2	11.3	0.6
*Stenella caeruleoalba*	Striped dolphin	5	−17.3	0.4	12.1	0.8
*Stromateus fiatola*	Blue butterfish	4	−17.3	0.3	10.8	0.2
*Tetrapturus belone*	Spearfish	5	−17.8	0.4	10.1	0.7
*Thunnus alalunga*	Albacore	5	−17.8	0.4	11.0	0.4
*Thunnus thynnus* >100 cm	Bluefin tuna	5	−18.3	0.3	10.3	0.6
*Thunnus thynnus* <100 cm	Bluefin tuna	5	−17.7	0.4	10.6	0.3
*Trachinotus ovatus*	Pompano	5	−17.5	0.4	11.2	0.3
*Trachurus trachurus* *	Horse mackerel	5	−17.6	0.2	10.5	0.5
*Xiphias gladius* >100 cm	Swordfish	5	−17.8	0.3	11.4	0.4
*Xiphias gladius* <50 cm	Swordfish	5	−17.8	0.7	11.2	0.2

* : *considered also as prey*; A: *colective samples*.

**Figure 2:**
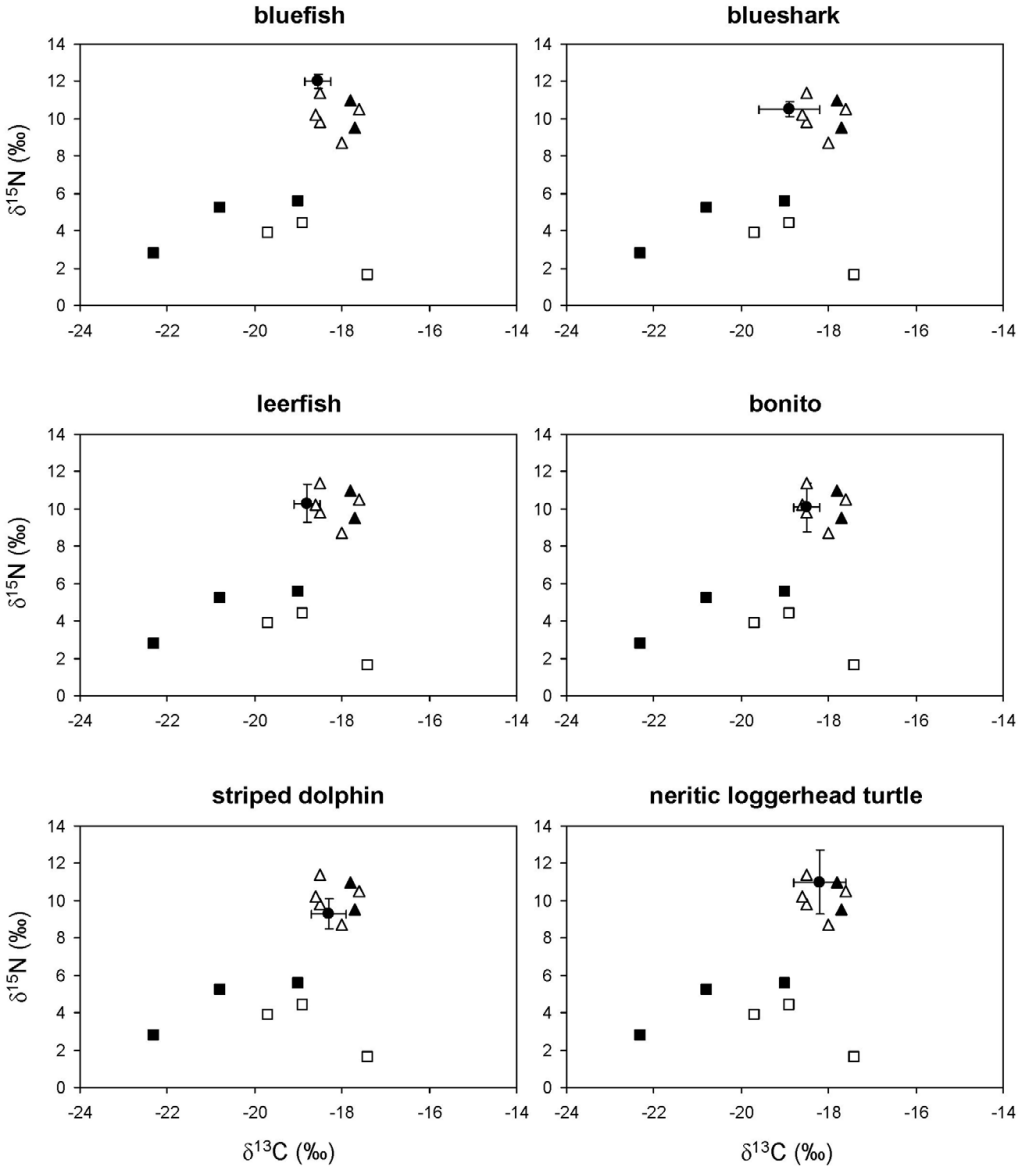
Stable isotope ratios of bluefish, blueshark, leerfish, bonito, striped dolphins and neritic loggerhead sea turtles from the northwestern Mediterranean. Solid circles represent the average stable isotope ratios of each consumer after correcting for diet-tissue isotopic discrimination and error bars show standard deviation. Other symbols show the average stable isotope ratios of potential prey: pelagic crustaceans (solid squares), gelatinous plankton (empty squares), squid (solid triangles) and small pelagic and mesopelagic fish (empty triangles).

**Figure 3:**
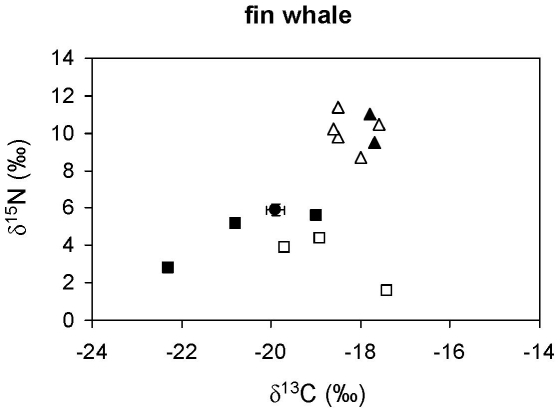
Stable isotope ratios of fin whales from the northwestern Mediterranean. A solid circle represents the average stable isotope ratios of whales after correcting for diet-tissue isotopic discrimination and error bars show standard deviation. Other symbols show the average stable isotope ratios of potential prey: pelagic crustaceans (solid squares), gelatinous plankton (empty squares), squid (solid triangles) and small pelagic and mesopelagic fish (empty triangles).

The ratios of stable isotopes in bluefish, blue shark, leerfish, bonito, striped dolphins and neritic loggerhead sea turtles (Figure 2) were consistent with the fish- and squid-dominated diet suggested by stomach content analysis (Table 2). Likewise, the ratio of stable isotopes in fin whales (Figure 3) was consistent with a crustacean-based diet (Table 2), although euphausiids were unlikely to be the only crustaceans consumed.

**Table 2:** Relative importance of gelatinous plankton in the diet of pelagic mesopredators and apex predators from the Mediterranean Sea, as revealed by stomach content analysis.

Species	Common name	Diet	References	
Auxis rochei	Bullet tuna	F,C,E,H,(U),(Cn)	Mostarda et al. 2007	[23]
*Balaenoptera physalus*	Fin whale	E	Laran et al. 2010	[55]
*Caretta caretta*	Loggerhead turtle	F,C,(U)	Tomás et al. 2001	[24]
			Revelles et al. 2007	[25]
*Coryphaena hippurus*	Dolphinfish	F,D,H,C,(Cn)	Massutí et al. 1998	[26]
*Euthynnus alletteratus*	Little tunny	F,C	Kyrtatos 1982	[56]
			Falautano et al. 2007	[67]
*Lichia amia*	Leerfish	F	Bennett 1989*	[57]
*Pomatomus saltatrix*	Bluefish	F,C	Buckel et al. 1999*	[60]
*Prionace glauca*	Blue shark	C,Ct,F	Henderson et al. 2001*	[61]
*Sarda sarda*	Atlantic bonito	F,(U)	Kyrtatos 1982	[56]
			Campo et al. 2006	[27]
*Scomber scombrus*	Mackerel	F,E,H	Kyrtatos 1982	[56]
*Seriola dumerili*	Amberjack	F,C,E	Matallanas et al. 1995	[69]
*Stenella caeruleoalba*	Striped dolphin	C, F	Blanco et al. 1995	[58]
			Meotti and Podestà1997	[59]
			Özturk et al. 2007	[62]
*Tetrapturus belone*	Spearfish	F,C,(U),(Cn)	Castriota et al. 2008	[28]
			Romeo et al. 2009	[31]
*Thunnus alalunga*	Albacore	F,H,E,C,U,(Cn)	Consoli et al. 2008	[29]
*Thunnus thynnus*	Bluefin tuna	F,C,D	Morovic 1961	[63]
			Kyrtatos 1982	[56]
			Orsi Relini et al. 1995	[66]
			Sanz Brau 1990	[64]
			Sinopoli et al. 2004	[30]
*Trachurus trachurus*	Horse mackerel	E, F	Ben Salem 1988	[68]
*Xiphias gladius*	Swordfish	F, C,(U),(Cn)	Chalabi and Ifrene 1992	[65]
			Orsi Relini et al. 1995	[66]
			Romeo et al. 2009	[31]

The diet column reports the preys contributing at least 5% in weight or volume to stomach contents (F: Teleostei; D: Decapoda, H: Hyperiidea, E: Euphausiids; C: Cephalopoda, Cn: Cnidaria, Ct: Cetaceans; U: Urochordata). Consumption of cnidarians and urochordata representing less than 5% is reported in brackets.

* *: data from the Atlantic*.

**Figure 4:**
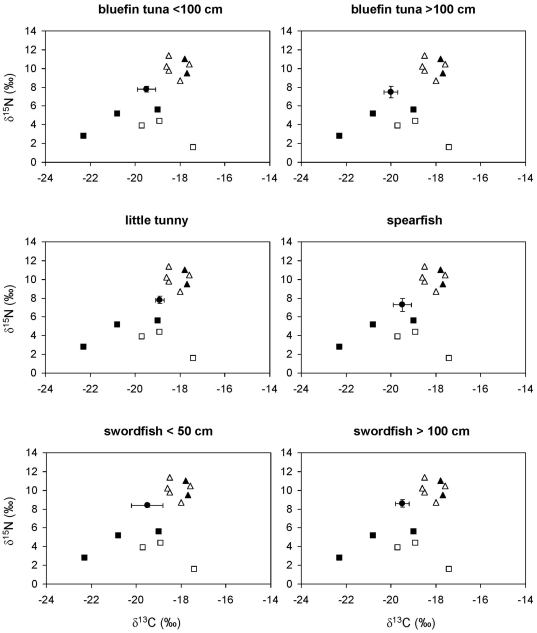
Stable isotope ratios of bluefin tuna, little tunny, spearfish, and swordfish from the northwestern Mediterranean. Solid circles represent the average stable isotope ratios of each consumer after correcting for diet-tissue isotopic discrimination and error bars show standard deviation. Other symbols show the average stable isotope ratios of potential prey: pelagic crustaceans (solid squares), gelatinous plankton (empty squares), squid (solid triangles) and small pelagic and mesopelagic fish (empty triangles).

**Figure 5:**
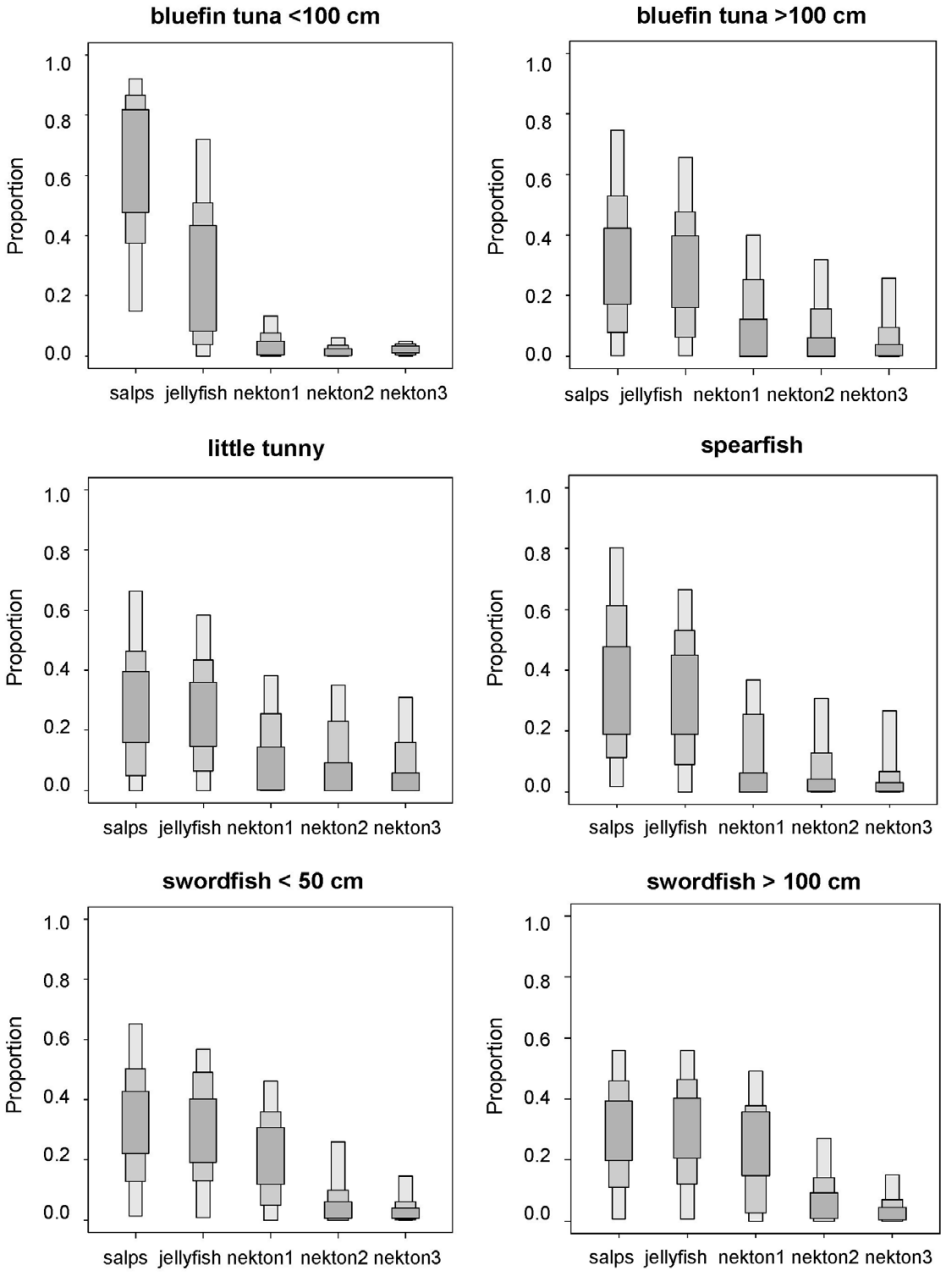
Feasible contribution of potential prey to the diet of bluefin tuna, little tunny, spearfish and swordfish according to SIAR. Nekton 1: sardine. Nekton 2: anchovy, lanternfish, horse mackerel and longfin squid. Nekton 3: mackerel and shortfin squid. Results are shown as 95, 75 and 25% credibility intervals for each prey.

**Figure 6:**
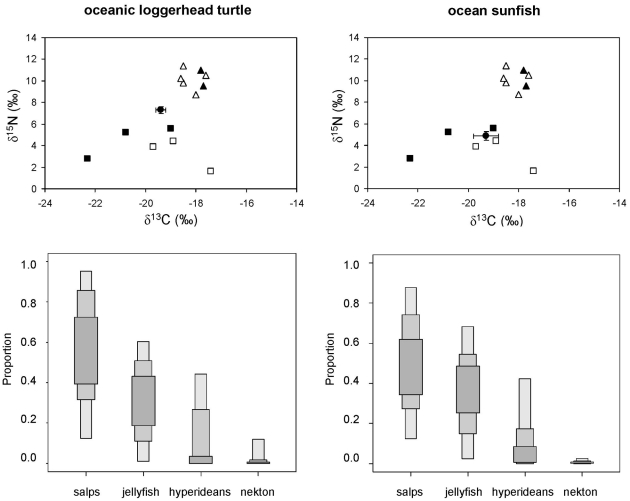
Stable isotope ratios of oceanic loggerhead sea turtle and ocean sunfish from the northwestern Mediterranean and feasible contribution of potential prey to their diet according to SIAR. Solid circles represent the average stable isotope ratios of each consumer after correcting for diet-tissue isotopic discrimination and error bars show standard deviation. Other symbols show the average stable isotope ratios of potential prey: pelagic crustaceans (solid squares), gelatinous plankton (empty squares), squid (solid triangles) and small pelagic and mesopelagic fish (empty triangles). Nekton: anchovy, lanternfish, horse mackerel and shortfin squid. Results are shown as 95, 75 and 25% credibility intervals for each prey.

In contrast, the ratios of stable isotopes in bluefin tuna, little tunny, spearfish and swordfish (Figure 4) were inconsistent with the fish- and squid-based diet suggested by stomach content analysis (Table 2). On the contrary, SIAR suggested a major role for gelatinous zooplankton in the diet of these four species (Figure 5), although there was a high uncertainty about the relative contribution of salps and pink jellyfish. It should be kept in mind that any esteem of the actual contribution of gelatinous zooplankton to the diet of these species could be affected by the uncertainty about the actual diet-tissue fractionation factors. Accordingly, the sensitivity analysis revealed that the mean contribution of salps to the diet of bluefin tuna larger than 100 cm could range from 30% to 58% and that of pink jellyfish from 29% to 31%, depending on the diet-tissue fractionation factors introduced into the model. Similar results were forum for bluefin tuna smaller than 100 cm. The ratios of stable isotopes in ocean sunfish and loggerhead sea turtles in the oceanic stage were also consistent with a jellyvorous diet, a result confirmed by SIAR (Figure 6).

**Figure 7:**
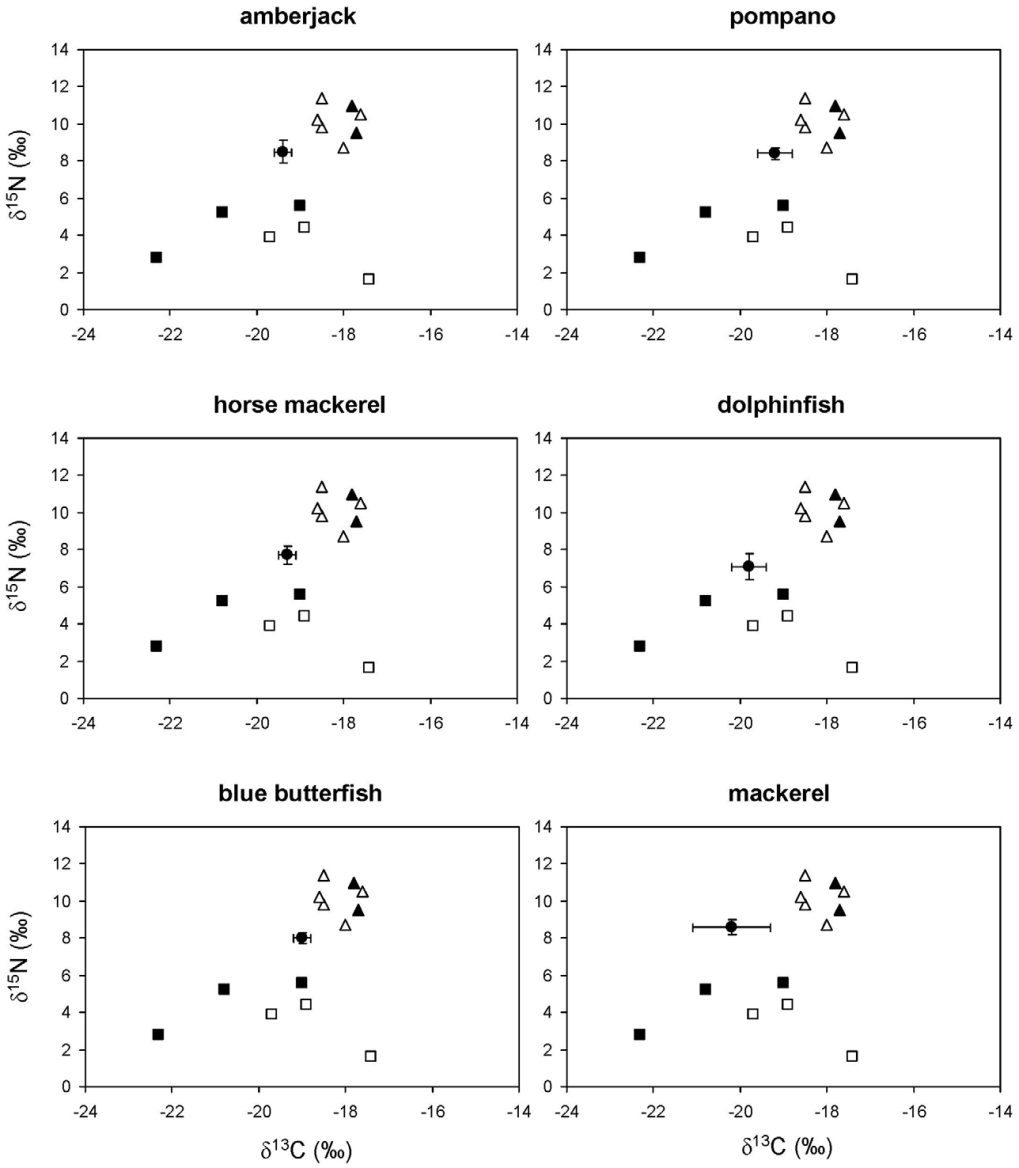
Stable isotope ratios of the diet of amberjack, pompano, horse mackerel, dolphinfish, blue butterfish and mackerel from the northwestern Mediterranean. Solid circles represent the average stable isotope ratios of each consumer after correcting for diet-tissue isotopic discrimination and error bars show standard deviation. Other symbols show the average stable isotope ratios of their potential prey: pelagic crustaceans (solid squares), gelatinous plankton (empty squares), squid (solid triangles) and small pelagic and mesopelagic fish (empty triangles).

**Figure 8:**
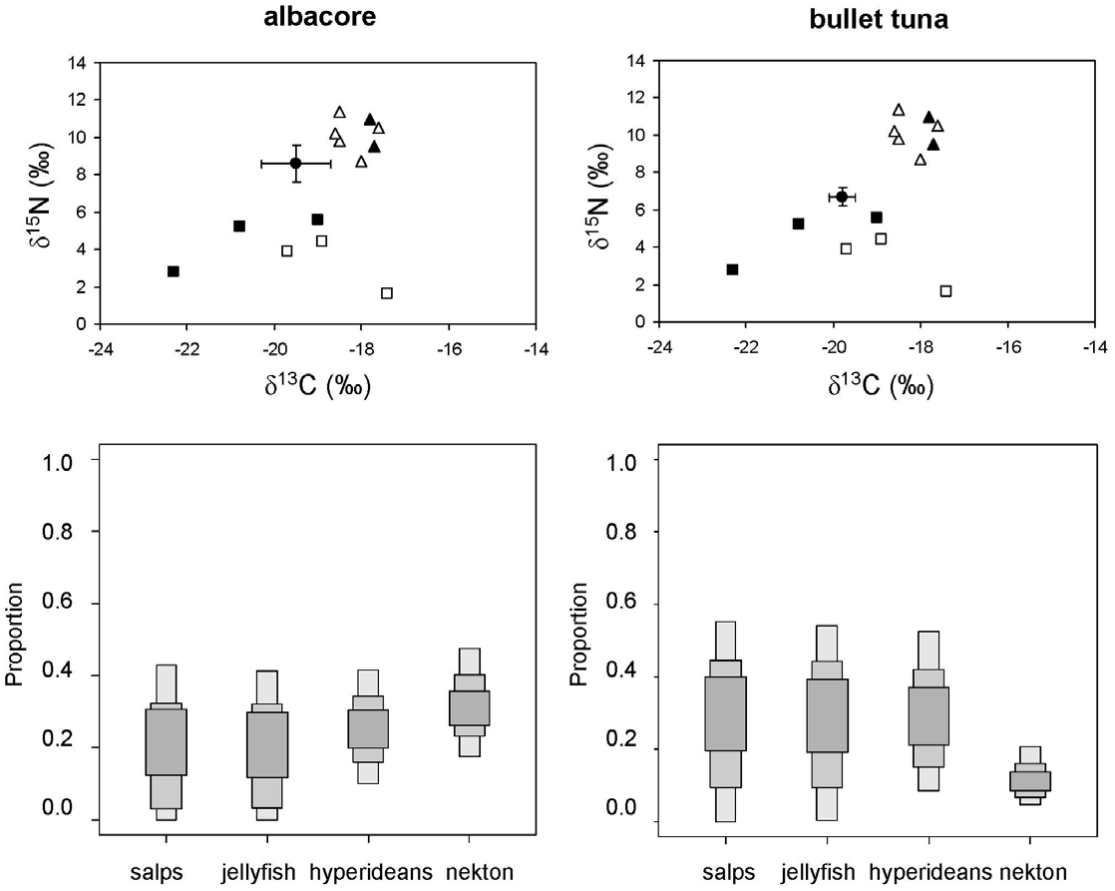
Stable isotope ratios of albacore and bullet tuna from the northwestern Mediterranean and feasible contribution of potential prey to their diet according to SIAR. Solid circles represent the average stable isotope ratios of each consumer after correcting for diet-tissue isotopic discrimination and error bars show standard deviation. Other symbols show the average stable isotope ratios of their potential prey: pelagic crustaceans (solid squares), gelatinous plankton (empty squares), squid (solid triangles) and small pelagic and mesopelagic fish (empty triangles). Nekton: anchovy, lanternfish, horse mackerel and shortfin squid. Results are shown as 95, 75 and 25% credibility intervals for each prey.

**Figure 9:**
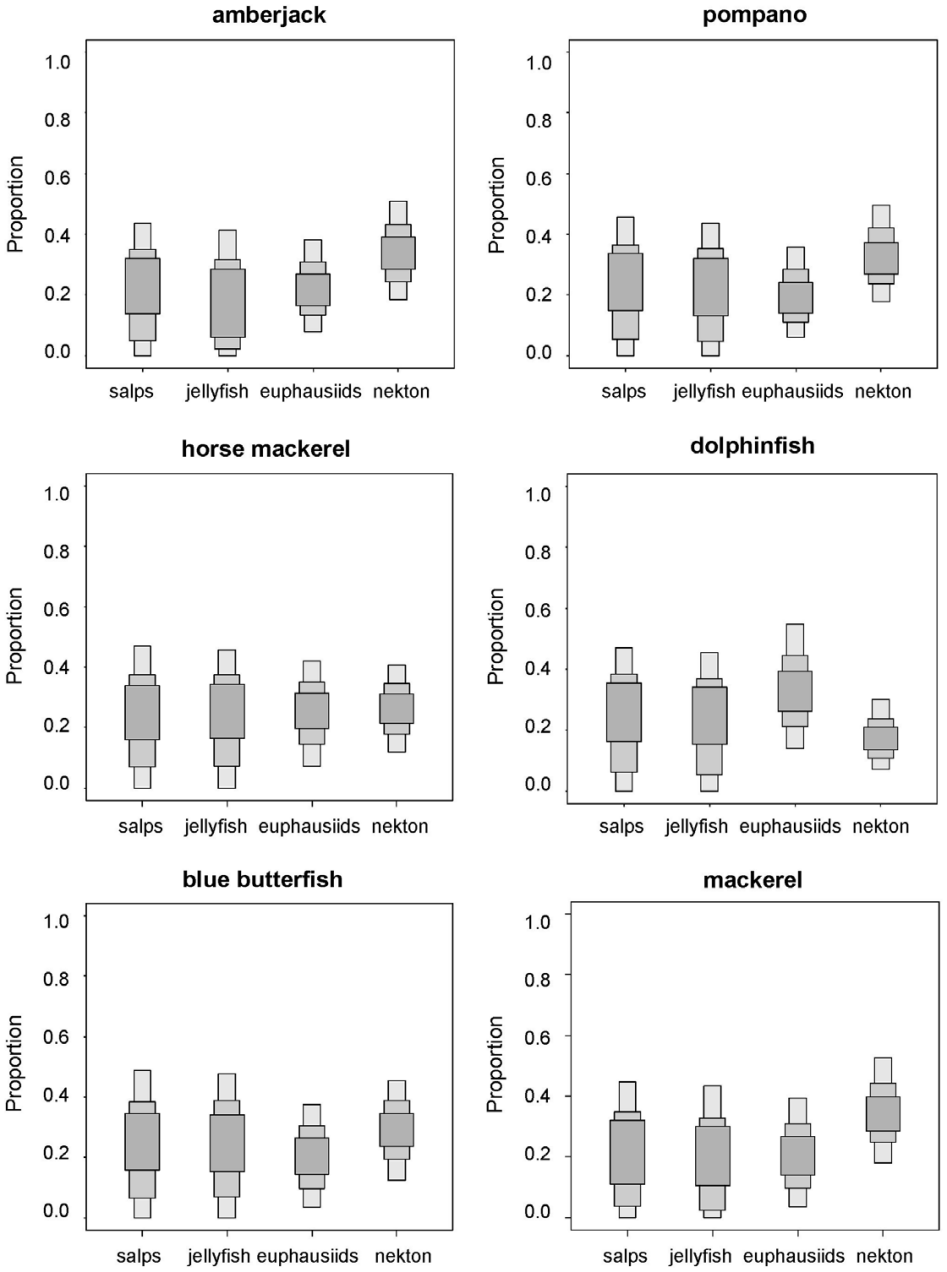
Feasible contribution of potential prey to the diet of amberjack, pompano, horse mackerel, dolphinfish, blue butterfish and mackerel according to SIAR. Nekton: anchovy, lanternfish, horse mackerel and longfin squid. Results are shown as 95, 75 and 25% credibility intervals for each prey.

The concentration of stable isotopes in the remaining species suggested diets with varying combinations of fishes, cephalopods and crustaceans (Figures 7 and 8), consistent with the results of stomach content analysis (Table 2). Nevertheless, SIAR was ambiguous about the relevance of salps and pink jellyfish in the diets of these species because, although the feasible contributions were similar to those of crustaceans, the credibility intervals were extremely loose (Figures 8 and 9).

The proximate chemical composition and energy density of the considered potential prey are shown in table 3. As expected, the energy density of mackerel was much higher that that of longfin squid, with in turn was higher than that of pink jellyfish and salps.

**Table 3:** Proximate chemical composition and energy density of four potential preys.

	Pink jellyfish	Salp	Mackerel	Longfin squid
**Sample size**	5	5	5	5
**Wet weight (g)**	42±9	19±14	248±31	152±23
**Water (%)**	96.3±0.1	95.8±0.5	72.4±0.5	81.3±0.4
**Ash (%)**	3.4±0.1	3.6±0.5	2.8±0.3	2.2±0.2
**Protein (%)**	0.2±0.1	0.2±0.1	12.3±0.4	13.2±0.3
**Fat (%)**	0.9±0.1	1.0±0.2	13.2±0.2	3.3±0.2
**Energy (kJ g_−1_)**	0.41±0.1	0.43±0.1	8.4±0.5	5.2±0.8

*Results are reported as mean ± standard deviation on a wet mass basis.*

## Discussion

The use of stable isotopes for dietary studies relies on three major assumptions. First, that isotope fractionation from prey to predator is known. Fractionation is species and stage specific and controlled experiments in captivity are the best method to calculate diet-tissue isotopic discrimination factors. This type of experimental data were available only for the loggerhead sea turtle [49], so for other fishes and mammals this study used previously reported average diet-tissue isotopic discrimination factors [50]. The sensitivity analysis revealed that the global contribution of gelatinous zooplankton to the diet was only slightly affected by the diet-tissue isotopic discrimination factors entered into the model, although the actual partitioning between salps and pink jellyfish was more sensitive.

The second assumption is that the variability in the ratios of stable isotopes of the potential prey is not obscured by migration between contrasting isoscapes. The western Mediterranean and the adjoining Atlantic differ in their isotopic baselines [51], and at least bluefin tuna and bullet tuna migrate annually between the two basins, moving into the Mediterranean in spring for spawning [34], [52]. However, the turnover of stable isotopes in the muscle of warm water fish is fast enough to capture changes in the stable isotope ratios of the diet in just a few months [38], [53], [54]. As the samples for the present study were collected from July to September, the stable isotope ratios reported here should reflect feeding in the Mediterranean. On the other hand, as isotope ratios in muscle integrate the diet over several months [38], [53], [54], the result here reported reflect dietary preferences over that time window and are not affected by short pulses of high food availability.

The third major assumption is that differences in the concentration of stable isotopes in the potential prey are large enough to allow proper discrimination among potential prey. Although statistically significant differences existed between all the species of macrozooplankton considered in the present study, there was considerable overlap in their ranges, as was also true for nekton. As a consequence, the performance of SIAR in resolving diet breakup within those two groups was often poor. However, for several species, the results were unambiguous when the ratios of stable isotope were combined with published information about stomach contents.

On this ground, seven of the species considered here are unlikely to consume relevant amounts of gelatinous plankton: bluefish, blue shark, bonito, fin whales, leerfish, loggerhead sea turtles (in the neritic stage) and striped dolphins. Although detailed studies on the stomach contents of Mediterranean fin whales are missing, these cetaceans are thought to rely primarily on crustaceans [55], a hypothesis supported by the ratios of stable isotopes reported here. Fish and squid dominate the stomach contents of bluefish, blue shark, leerfish and striped dolphins [24], [27], [56]–[62], although low numbers of salps have been reported from the stomach contents of bonito [27] and neritic loggerhead sea turtles [24]. Nevertheless, the concentrations of stable isotopes in all of these species were highly consistent with a nektonic diet, and no doubt exists that gelatinous plankton play no relevant role in their diets.

Fish and squid also dominate the stomach contents of bluefin tuna, little tunny, swordfish and spearfish [28], [30], [31], [56], [63]–[67], but all of these species are highly depleted in ^15^N when compared with the fish and cephalopod consumers reported above and with their potential prey. Estrada et al. (2005) [53] reported a similar depletion for tuna in the northwestern Atlantic and attributed it to the overlooked consumption of some other type of unidentified zooplankton. The δ^15^N of decapods is close to that of zooplanktophagous fish [38], [54], and hence, their consumption cannot cause the depletion of ^15^N reported here. Euphausiids and hyperiideans are more depleted in ^15^N than fish (this study), but there is no reason for them to be overlooked in dietary studies, as they have been found in large numbers in the stomach contents of other species (Table 2). Thus, gelatinous plankton is the most likely source of ^15^N depleted food for bluefin tuna, little tunny, swordfish and spearfish and, according to SIAR, represents a significant fraction of their diets.

Albacore, mackerel, bullet tuna, dolphinfish, amberjack and horse mackerel also consume fishes and squids, but crustaceans are relatively abundant in their stomach contents (Table 2), which may explain why they are more depleted in ^15^N than pure nekton consumers. Nevertheless, the consumption of gelatinous plankton cannot be completely ruled out, as salps and jellyfishes occur in low numbers in the stomach contents of at least some of these species (Table 2). The diet of the blue butterfish has not been investigated in detail in the Mediterranean, but the blue butterfish is thought to consume fishes, crustaceans and jellyfishes elsewhere [41]. The inspection of the stomach contents of the individuals collected for this study revealed fish remnants mixed with a purplish paste reminiscent of pink jellyfish tissue, although the δ^15^N values were too high to be indicative of a diet based on gelatinous plankton.

Finally, stable isotopes confirmed the reliance of oceanic loggerhead sea turtles and ocean sunfish on gelatinous plankton. The differences in the ratios of stable isotopes of oceanic and neritic loggerhead sea turtles reported here are consistent with the satellite telemetry data reported by Cardona et al. (2009) [70], revealing the existence of two well-delineated groups of loggerhead sea turtles off mainland Spain with contrasting patterns of habitat use. This explains the dramatic differences observed in the isotope ratios of the loggerhead sea turtles captured on-shore and off-shore mainland Spain. The situation is completely different off the Balearic Islands, where true neritic turtles do not exist [71], [72], and no major differences have been observed in the isotope ratios of turtles captured over the continental shelf and off-shore [39].

The overall evidence presented here suggests the existence of a guild of gelatinous plankton consumers including two specialists (ocean sunfish and loggerhead sea turtles in the oceanic stage) and several opportunists (bluefin tuna, little tunny, spearfish and swordfish. However, some further calculations are needed to demonstrate that massive consumption of gelatinous zooplankton by these species is energetically possible, considering the low energy density of gelatinous plankton (Table 3), the large body mass of most of the gelatinous consumers and their food consumption rates [73]–[76].

The daily ration of captive bluefin tuna fed with fishes and squids ranges from 4.3% to 1.5% body mass, depending on tuna size [76]. Assuming that the energy density of a mixed diet including fishes and squids is 6.8 kJ g^−1^ (Table 3), the individual daily energy intake of a small bluefin tuna (15 kg) is 4,386 kJ and that of a large bluefin tuna (100 kg) is 20,400 kJ. According to SIAR, gelatinous zooplankton may represent as much as 80% of the diet of small bluefin tuna and 60% of that of large bluefin tuna. To meet these proportions, a small bluefin tuna (15 kg) should eat daily 0.13 kg of fishes and squids and 8.5 kg of gelatinous zooplankton with an energy content of 3,509 kJ, equivalent to 270 pink jellyfish (Table 3). Likewise, a large bluefin tuna (100 kg) should eat daily 0.60 kg of fishes and squids and 14.2 kg of gelatinous zooplankton with an energy content of 6,120 kJ, equivalent to 474 pink jellyfish (Table 3). However, SIAR results have wide credibility intervals, so is possible that the consumption of gelatinous zooplankton by bluefin tuna is lower. For instance, if gelatinous zooplankton represents 60% and 30% of the diet of small and large bluefin tuna respectively, they should eat daily 6.3 kg and 7.1 kg of gelatinous zooplankton respectively.

These quantities may seem large, but the biomass of gelatinous zooplankton in the epipelagic region of the Mediterranean Sea ranges usually 1–10 kg 100 m^−3^, with the biomass of the pink jellyfish reaching sometimes values as high as 24 kg 100 m^−3^ [10]. This means that a bluefin tuna picking effortless jellyfish as it encounter them can satisfy its daily energy requirements after swimming just a few hundred meters across a swarm of gelatinous plankton. However, this tuna will probably not be able to swallow the required biomass of jellyplankton in a single meal, so more or less continuous consumption of gelatinous plankton through light hours is a more likely scenario.

The results here reported demonstrate the plausibility that top predators control the abundance of gelatinous zooplankton, but do not prove it. Further research is needed to confirm that bluefin tuna, little tunny, spearfish and swordfish consume large amounts of gelatinous plankton across the Mediterranean. Stable isotope ratios from different regions and years with contrasting abundance of gelatinous zooplankton will be extremely useful as confirmatory evidence. The use of other intrinsic tracers, like fatty acids, can also be useful to precise the proportion of gelatinous in the diet of these species and perhaps would help to better resolve the consumption of gelatinous zooplankton by species like mackerel, bullet tuna or dolphinfish. Behavioral observations of tuna as they swim across jellyfish swarms will also be extremely helpful to understand how gelatinous plankton is handled and consumed. And last, but not least, detailed data on the demography of gelatinous zooplankton are urgently needed to allow modeling how the depletion of top predators might have be caused, together with climate forcing, recent jellyfish outbreaks.
